# Identification of high-performing antibodies for SPARC-related modular calcium-binding protein 1 (SMOC-1) for use in Western Blot and immunoprecipitation

**DOI:** 10.12688/f1000research.141800.2

**Published:** 2024-09-05

**Authors:** Riham Ayoubi, Sara González Bolívar, Michael Nicouleau, Kathleen Southern, Carl Laflamme, NeuroSGC/YCharOS/EDDU collaborative group

**Affiliations:** 1 Department of Neurology and Neurosurgery, Structural Genomics Consortium, The Montreal Neurological Institute, McGill University, Montreal, Québec, H3A 2B4, Canada; 2 The Neuro’s Early Drug Discovery Unit (EDDU), Structural Genomics Consortium, The Montreal Neurological Institute, McGill University, Montreal, Québec, H3A 2B4, Canada

**Keywords:** Uniprot ID Q9H4F8, SMOC1, SMOC-1, SPARC-related modular calcium binding protein 1, antibody characterization, antibody validation, western blot, immunoprecipitation

## Abstract

SPARC-related modular calcium-binding protein 1, otherwise known as SMOC-1, is a secreted glycoprotein involved in various cell biological processes including cell-matrix interactions, osteoblast differentiation, embryonic development, and homeostasis. SMOC-1 was found to be elevated in asymptomatic Alzheimer’s disease (AD) patient cortex as well as being enriched in amyloid plaques and in AD patientcerebrospinal fluid, arguing for SMOC-1 as a promising biomarker for AD. Having access to high-quality SMOC-1 antibodies is crucial for the scientific community. It can ensure the consistency and reliability of SMOC-1 research, and further the exploration of its potential as both a therapeutic target or diagnostic marker.. In this study, we characterized seven SMOC-1 commercial antibodies for Western blot and immunoprecipitation, using a standardized experimental protocol based on comparing read-outs in knockout cell lines and isogenic parental controls. We identified successful antibodies in the tested applications and encourage readers to use this report as a guide to select the most appropriate antibody for their specific needs.

## Introduction

The *SMOC1* gene encodes the SPARC (secreted protein acidic and rich in cysteine)-related calcium-binding protein 1 (SMOC-1), a secreted glycoprotein involved in numerous extracellular processes. ^1^^–^^3^ Expressed in various tissues with localization to the basement membrane and extracellular matrix, SMOC-1 regulates cell-matrix interactions through its ability to bind cell-surface receptors, growth factors, extracellular matrix and cytokines. ^2^^,^^4^ Through its binding to receptors on the cells surface, SMOC-1 modulates growth factor signalling involved in osteoblast differentiation. ^5^ In addition to being a critical regulator of various biological processes, SMOC-1 plays a role in the pathophysiology of diverse diseases, including cancer development and progression. ^1^

Proteomic studies have uncovered SMOC-1 to be highly enriched in a subpopulation of amyloid plaques, in AD patients and to be elevated in asymptomatic AD cortex. ^6^ Recently, SMOC-1 was shown to be elevated in cerebrospinal fluid from AD patients. ^7^ Although it remains unknown why SMOC-1 co-localizes with only some amyloid plaques, it is hypothesized that SMOC-1 may interact with amyloid-beta (Aβ) species that have been subjected to post-translational modifications. ^6^ More comprehensive research is required to examine the mechanistic role of SMOC-1 in AD.

Mechanistic studies would be greatly facilitated with the availability of high-quality antibodies.

Here we evaluated the performance of seven commercial antibodies for SMOC-1 for use in western blot and immunoprecipitation, enabling biochemical and cellular assessment of SMOC-1 properties and function. The platform for antibody characterization used to carry out this study was endorsed by a committee of industry academic representatives. It consists of identifying human cell lines with adequate target protein expression and the development/contribution of equivalent knockout (KO) cell lines, followed by antibody characterization procedures using most commercially available antibodies against the corresponding protein. The standardized consensus antibody characterization protocols are openly available on Protocol Exchange (DOI: 10.21203/rs.3.pex-2607/v1). ^8^

The authors do not engage in result analysis or offer explicit antibody recommendations. Our primary aim is to deliver top-tier data to the scientific community, grounded in Open Science principles. This empowers experts to interpret the characterization data independently, enabling them to make informed choices regarding the most suitable antibodies for their specific experimental needs. Guidelines on how to interpret antibody characterization data found in this study are featured on the YCharOS gateway. ^9^

## Results and discussion

Our standard protocol involves comparing readouts from parental and knockout cells. ^10^^–^^14^ To identify a cell line that expresses adequate levels of SMOC-1 protein to provide sufficient signal to noise, we examined public proteomics databases, namely PaxDB ^15^ and DepMap. ^16^ HeLa was identified as a suitable cell line and thus HeLa was modified with CRISPR/Cas9 to knockout the corresponding *SMOC1* gene ( Table 1).

**Table 1.** Summary of the cell lines used.

Institution	Catalog number	RRID (Cellosaurus)	Cell line	Genotype
ATCC	CCL-2	CVCL_0030	HeLa	WT
Montreal Neurological Institute	-	CVCL_B7DT	HeLa	*SMOC1* KO

**Figure 1.**
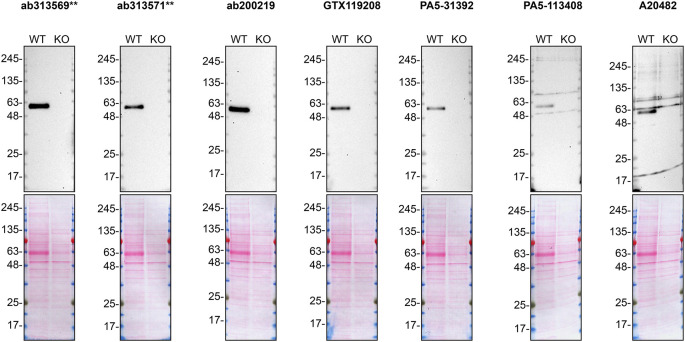
SMOC-1 antibody screening by Western blot on culture media. HeLa WT and *SMOC1* KO were cultured in serum free media, and 30 μg of protein from concentrated culture media were processed for Western blot with the indicated SMOC-1, antibodies. The Ponceau stained transfers of each blot are shown. Peroxidase-conjugated goat anti-rabbit was used as the secondary antibody to detect the signal produced. Antibody dilutions were chosen according to the recommendations of the antibody supplier. All antibodies were tested at 1/2000. Predicted band size: 48 kDa. **= recombinant antibody.

**Figure 2.**
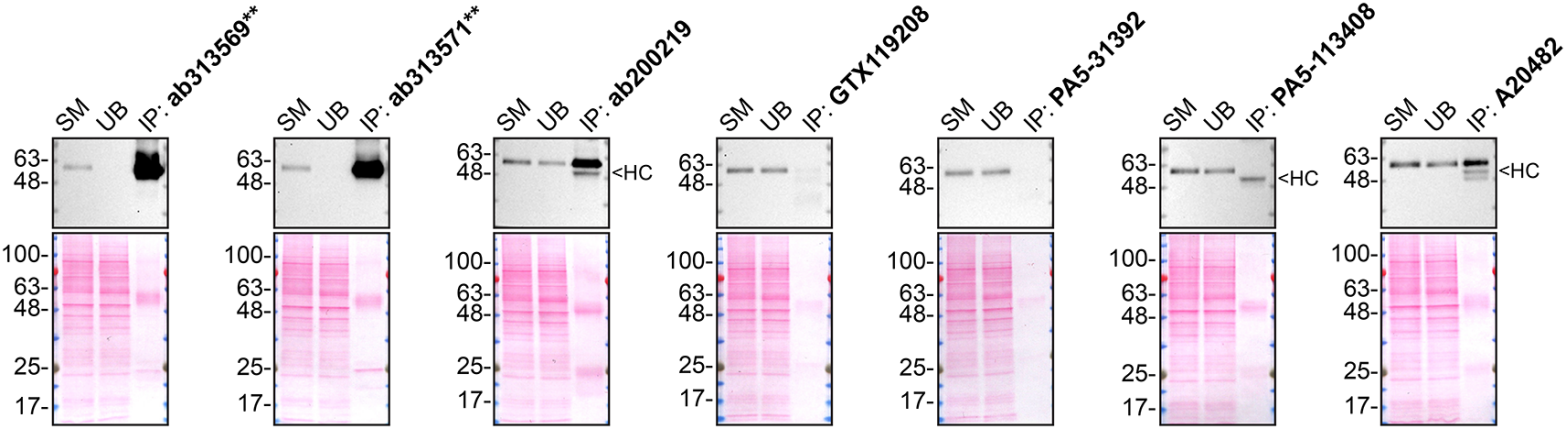
SMOC-1 antibody screening by immunoprecipitation on culture media. Immunoprecipitation was performed on concentrate culture media from HeLa WT, and using 2.0 μg of the indicated SMOC-1, antibodies pre-coupled to Dynabeads protein A. Samples were washed and processed for Western Blot with the indicated SMOC-1, antibody. For Western blot, ab313569** was used at 1/1000. The Ponceau stained transfers of each blot are shown for similar reasons as in Figure 1. VeriBlot for IP Detection Reagent:HRP was used as a secondary detection system. SM=8% starting material; UB=8% unbound fraction; IP=immunoprecipitated, HC= antibody heavy chain, **= recombinant antibody.

SMOC-1 is predicted to be a secreted protein. Accordingly, we collected concentrated culture media from both parental and *SMOC1* KO cells and used the conditioned media to probe the performance of the antibodies ( Table 2) side-by-side by Western blot and immunoprecipitation. The profiles of the tested antibodies are shown in Figures 1 and 2.

**Table 2.** Summary of the SMOC-1 antibodies tested.

Company	Catalog number	Lot number	RRID (Antibody Registry)	Clonality	Clone ID	Host	Concentration (μg/μL)	Vendors recommended applications
Abcam	ab313569 **	3101091230	AB_2941846 ^1^	recombinant-mono	EPR26922-29	rabbit	0.50	Wb, IP, IF
Abcam	ab313571 **	3101065175	AB_2941847 ^1^	recombinant-mono	EPR26922-31	rabbit	0.50	WB, IP
Abcam	ab200219	GR3370372-1	AB_2833001	polyclonal	-	rabbit	0.50	Wb
GeneTex	GTX119208	40331	AB_10618293	polyclonal	-	rabbit	0.90	Wb
Thermo Fisher Scientific	PA5-31392	130141931	AB_2548866	polyclonal	-	rabbit	0.90	Wb
Thermo Fisher Scientific	PA5-113408	WL3463969	AB_2868141	polyclonal	-	rabbit	3.50	Wb
ABclonal	A20482	125410101	AB_2909795	polyclonal	-	rabbit	2.65	Wb

Wb=Western blot; IF= immunofluorescence; IP=immunoprecipitation.

** = recombinant antibody.

^1^ refers to new antibodies with RRID that have recently been created (August 2023) but will be available on the Antibody Registry in the coming weeks.

In conclusion, we have screened seven SMOC-1 commercial antibodies by Western blot and immunoprecipitation. Under our standardized experimental conditions, several high-quality antibodies were identified, however, the authors do not engage in result analysis or offer explicit antibody recommendations. A limitation of this study is the use of universal protocols - any conclusions remain relevant within the confines of the experimental setup and cell line used in this study. Our primary aim is to deliver top-tier data to the scientific community, grounded in Open Science principles. This empowers experts to interpret the characterization data independently, enabling them to make informed choices regarding the most suitable antibodies for their specific experimental needs.

The underlying data can be found on Zenodo, an open-access repository. ^17^^,^^18^

## Methods

### Antibodies

All SMOC-1, antibodies are listed in Table 2, together with their corresponding Research Resource Identifiers (RRID), to ensure the antibodies are cited properly. ^19^ Peroxidase-conjugated goat anti-rabbit is from Thermo Fisher Scientific (cat. number 65-6120).

### CRISPR/Cas9 genome editing

HeLa *SMOC1* KO clone was generated with low passage cells using an open-access protocol available on Zenodo.org. The guide RNA used to knockout the *SMOC1* gene is CUCGUAGGACCUGCCAUCAG.

### Cell culture

Both HeLa WT and *SMOC1* KO cell lines used are listed in Table 1, together with their corresponding RRID, to ensure the cell lines are cited properly. ^20^ Cells were cultured in DMEM high-glucose (GE Healthcare cat. number SH30081.01) containing 10% fetal bovine serum (Wisent, cat. number 080450), 2 mM L-glutamate (Wisent cat. number 609065), 100 IU penicillin and 100 μg/mL streptomycin (Wisent cat. number 450201). Cells were starved in DMEM high-glucose containing L-glutamate and penicillin/streptomycin.

### Antibody screening by Western blot on culture media

HeLa cells WT and *SMOC1* KO were washed three times with PBS 1x and starved for ~18 hrs. Culture media were collected and centrifuged for 10 min at 500 x g to eliminate cells and larger contaminants, then for 10 min at 4500 x g to eliminate smaller contaminants. Culture media were concentrated by centrifuging at 4000 x g for 30 min using Amicon Ultra-15 Centrifugal Filter Units with a membrane NMWL of 10 kDa (MilliporeSigma cat. number UFC901024). Culture media were supplemented with 1x protease inhibitor cocktail mix (MilliporeSigma, cat. number P8340).

Western blots were performed as described in our standard operating procedure. ^12^^–^^14^^,^^21^ Western blots were performed with precast midi 4-20% Tris-Glycine polyacrylamide gels from Thermo Fisher Scientific (cat. number WXP42012BOX) ran with Tris/Glycine/SDS buffer from Bio-Rad (cat. number 1610772), loaded in Laemmli loading sample buffer from Thermo Fisher Scientific (cat. number AAJ61337AD) and transferred on nitrocellulose membranes. BLUelf prestained protein ladder from GeneDireX (cat. number PM008-0500) was used. Proteins on the blots were visualized with Ponceau S staining (Thermo Fisher Scientific, cat. number BP103-10) which is scanned to show together with individual Western blot. Blots were blocked with 5% milk for 1 hr, and antibodies were incubated overnight at 4°C with 5% milk in TBS with 0,1% Tween 20 (TBST) (Cell Signalling Technology, cat. number 9997). Following three washes with TBST, the peroxidase conjugated secondary antibody was incubated at a dilution of ~0.2 μg/ml in TBST with 5% milk for 1 hr at room temperature followed by three washes with TBST. Membranes were incubated with Pierce ECL from Thermo Fisher Scientific (cat. number 32106) prior to detection with the iBright™ CL1500 Imaging System from Thermo Fisher Scientific (cat. number A44240).

### Antibody screening by immunoprecipitation on culture media

Immunoprecipitation was performed as described in our standard operating procedure. ^12^^–^^14^^,^^22^ Antibody-bead conjugates were prepared by adding 2 μg of antibody to 500 μL of Pierce IP Lysis Buffer from Thermo Fisher Scientific (cat. number 87788) in a 1.5 mL microcentrifuge tube, together with 30 μL of Dynabeads protein A- (for rabbit antibodies) from Thermo Fisher Scientific (cat. number 10002D). Tubes were rocked for ~1 hr at 4°C followed by two washes to remove unbound antibodies.

Starved HeLa WT culture media were concentrated as described above and supplemented with protease inhibitor. 0.3 mL aliquots at 1.6 mg/mL of protein were incubated with an antibody-bead conjugate for ~1 hr at 4°C. The unbound fractions were collected, and beads were subsequently washed three times with 1.0 mL of IP lysis buffer and processed for SDS-PAGE and Western blot on a precast midi 4-20% Tris-Glycine polyacrylamide gels. VeriBlot for IP Detection Reagent:HRP from Abcam (cat. number ab131366) was used as a secondary detection system at a concentration of 0.3 μg/mL.

## Data Availability

Zenodo: Antibody Characterization Report for SMOC-1, https://doi.org/10.5281/zenodo.8277962. ^17^ Zenodo: Dataset for the SPARC-related modular calcium-binding protein 1 (SMOC-1) antibody screening study, https://doi.org/10.5281/zenodo.8253319. ^18^ Data are available under the terms of the Creative Commons Attribution 4.0 International license (CC-BY 4.0).
