# Supplementary material for: Identification of high-performing antibodies for SPARC-related modular calcium-binding protein 1 (SMOC-1) for use in Western Blot and immunoprecipitation
Source: F1000Res. 2024 Sep 5;12:1279. Originally published 2023 Oct 6. [Version 2] doi: 10.12688/f1000research.141800.2 (PMC11406118; doi:10.12688/f1000research.141800.2)
Supplement: Peer review history

# Reviewer report: Reviewer response for version 2

**DOI:** 10.5256/f1000research.171274.r321083

**Published:** 2026-02-27

**Authors:** Deborah Moshinsky

**Affiliations:** 1 Institute for Protein Innovation, Boston, Massachusetts, USA

No further comments

Are sufficient details of methods and materials provided to allow replication by others?

Yes

Is the rationale for creating the dataset(s) clearly described?

Yes

Are the datasets clearly presented in a useable and accessible format?

Yes

Are the protocols appropriate and is the work technically sound?

Yes

Reviewer Expertise:

antibody characterization and validation

I confirm that I have read this submission and believe that I have an appropriate level of expertise to confirm that it is of an acceptable scientific standard.

# Reviewer report: Reviewer response for version 2

**DOI:** 10.5256/f1000research.171274.r321084

**Published:** 2024-09-16

**Authors:** Christian Tiede

**Affiliations:** 1 University of Leeds, Leeds, England, UK

see previous report. The responses to the comments are satisfactory, except for point 3, which is still confusing and unclear.

Are sufficient details of methods and materials provided to allow replication by others?

Yes

Is the rationale for creating the dataset(s) clearly described?

Yes

Are the datasets clearly presented in a useable and accessible format?

Yes

Are the protocols appropriate and is the work technically sound?

Partly

Reviewer Expertise:

antibody alternative scaffolds, phage display

I confirm that I have read this submission and believe that I have an appropriate level of expertise to confirm that it is of an acceptable scientific standard.

# Reviewer report: Reviewer response for version 1

**DOI:** 10.5256/f1000research.155279.r313912

**Published:** 2024-08-30

**Authors:** Christian Tiede

**Affiliations:** 1 University of Leeds, Leeds, England, UK

In this manuscript, Ayoubi et al. investigated the specificity of seven commercial anti-SMOC1 antibodies in Western Blot and immunoprecipitation utilising HeLa wild-type and knockout cells. The manuscript is well-written, with experiments well executed and technically sound. Although the authors do not provide a detailed analysis and interpretation of their results, the data are well presented and clearly indicate suitable antibodies for WB and IP studies. Additional comments:

 1) It would have been helpful if the authors had explained why immunofluorescence or immunohistochemistry were not considered for this study.

 2) It would be useful to also indicate in the figure captions that a secondary antibody was used.

 3) It might be understandable that the authors do not make explicit antibody recommendations. However, the controversial result of the secondary reagent used for IP should be discussed. VeriBlot for IP detection reagent was most likely used to avoid interference from denatured IgG. Nevertheless, the heavy chain was detected in some antibodies.

 4) In the methods section on immunoprecipitation, the authors should clarify how samples were processed for SDS-PAGE and Western blotting.

Are sufficient details of methods and materials provided to allow replication by others?

Yes

Is the rationale for creating the dataset(s) clearly described?

Yes

Are the datasets clearly presented in a useable and accessible format?

Yes

Are the protocols appropriate and is the work technically sound?

Partly

Reviewer Expertise:

antibody alternative scaffolds, phage display

I confirm that I have read this submission and believe that I have an appropriate level of expertise to confirm that it is of an acceptable scientific standard, however I have significant reservations, as outlined above.

# Response

**Published:** 2024-08-30

**Authors:** Kathleen Southern

**Affiliations:** McGill University, Canada

Thank you Christian Tiede for your comprehensive report of this Data note. After reading your comments and feedback, we will be submitting an updated version of this article as to address your concerns and provide clarifications on specific experimental setups used. We are confident our response, as well as the new version submitted have clarified any concerns you may previously have had.

***1) It would have been helpful if the authors had explained why immunofluorescence or immunohistochemistry were not considered for this study.***

  Following our standardized antibody characterization platform, available on Protocol exchange ( https://doi.org/10.21203/rs.3.pex-2607/v1), immunofluorescence (IF) is not performed when the target protein is secreted. Given the fixation and permeabilization methods used in this protocol, performing IF for secreted proteins presents many limitations and challenges.

 Immunohistochemistry (IHC) is not currently within the characterization platform. The main reason being that, given our knockout (KO) cell line-based protocol, it would be extremely challenging to KO proteins in human tissues for the entire human genome. That being said, the idea of including IHC to the platform is being troubleshooted and we hope to include this antibody-based application in the future. We’ve recently included Flow Cytometry  (FC) to our protocol and are in the process of revisiting many Data Notes to include the FC data.

***2) It would be useful to also indicate in the figure captions that a secondary antibody was used.***

*Thank you for this feedback.*

 We will be submitting a new version of this manuscript and the secondary antibodies and detection systems used will be included in the figure legends for all applications.

***3) It might be understandable that the authors do not make explicit antibody recommendations. However, the controversial result of the secondary reagent used for IP should be discussed. VeriBlot for IP detection reagent was most likely used to avoid interference from denatured IgG. Nevertheless, the heavy chain was detected in some antibodies.***

 VeriBlot was used as a secondary detection system as the expected SMOC-1 molecular weight was very similar to that of the immunoglobulin heavy chain, 48 kDa and 50 kDa, respectively. The proximity of these molecular weights can cause antibodies used in the WB following the IP to cross-react with the heavy chain, preventing the true immunoprecipitated signal from being detected. That is why VeriBlot was used as the secondary detection systems as it would not react with the heavy chain. This is proven correct as the heavy chain is detected independently in the IP of 3 antibodies (ab200219, PA5-113408 and A20482).

 To understand our selection method of secondary detection systems for IP experiments, please refre to Figure 9 of the detailed procedure on Protocol exchange ( https://doi.org/10.21203/rs.3.pex-2607/v1), that will be listed as reference 8 in the updated version of the article.

***4) In the methods section on immunoprecipitation, the authors should clarify how samples were processed for SDS-PAGE and Western blotting.***

 In the updated version of this manuscript, to be submitted shortly, we will include the reference to our entire antibody characterization platform which provides the step-by-step details of this procedure.

# Reviewer report: Reviewer response for version 1

**DOI:** 10.5256/f1000research.155279.r314301

**Published:** 2024-08-27

**Authors:** Deborah Moshinsky

**Affiliations:** 1 Institute for Protein Innovation, Boston, Massachusetts, USA

SPARC (secreted protein acidic and rich in cysteine)-related calcium-binding protein 1 (SMOC-1) is involved in the pathophysiology of a number of diverse diseases.  In Alzheimer's disease (AD), SMOC-1 has been found to co-localize with some amyloid plaques, although it's mechanistic role in the disease has not been well-investigated.  The availability of high quality antibodies would facilitate research into SMOC-1's involvement in AD, so the investigators compared the performance of 7 commercially available antibodies in Western blot and  immunoprecipitation to allow researchers to assess antibody performance in these applications.

 The authors present a scientifically sound Western blot and immunoprecipitation study of all 7 antibodies against SMOC-1.  The protocols were presented in sufficient detail for the work to be repeated and the reader can interpret whether the antibodies worked or not under the given experimental conditions.  No assessment of the results was given, however the authors give appropriate reasoning for not including such an assessment.

Are sufficient details of methods and materials provided to allow replication by others?

Yes

Is the rationale for creating the dataset(s) clearly described?

Yes

Are the datasets clearly presented in a useable and accessible format?

Yes

Are the protocols appropriate and is the work technically sound?

Yes

Reviewer Expertise:

antibody characterization and validation

I confirm that I have read this submission and believe that I have an appropriate level of expertise to confirm that it is of an acceptable scientific standard.
